# Microstructured Thin Film Nitinol for a Neurovascular Flow-Diverter

**DOI:** 10.1038/srep23698

**Published:** 2016-03-24

**Authors:** Yanfei Chen, Connor Howe, Yongkuk Lee, Seongsik Cheon, Woon-Hong Yeo, Youngjae Chun

**Affiliations:** 1 Department of Industrial Engineering, University of Pittsburgh, Pittsburgh, PA 15261, USA; 2 Department of Mechanical and Nuclear Engineering, Virginia Commonwealth University, VA 23284, USA; 3 Division of Mechanical and Automotive Engineering, Kongju National University, Cheonan, Chungnam, 314-701, Republic of Korea; 4 Center for Rehabilitation Science and Engineering, Virginia Commonwealth University, VA 23298, USA; 5 Department of Bioengineering, University of Pittsburgh, PA 15261, USA

## Abstract

A cerebral aneurysm occurs as a result of a weakened blood vessel, which allows blood to flow into a sac or a ballooned section. Recent advancement shows that a new device, ‘flow-diverter’, can divert blood flow away from the aneurysm sac. People found that a flow-diverter based on thin film nitinol (TFN), works very effectively, however there are no studies proving the mechanical safety in irregular, curved blood vessels. Here, we study the mechanical behaviors and structural safety of a novel microstructured TFN membrane through the computational and experimental studies, which establish the fundamental aspects of stretching and bending mechanics of the structure. The result shows a hyper-elastic behavior of the TFN with a negligible strain change up to 180° in bending and over 500% in radial stretching, which is ideal in the use in neurovascular curved arteries. The simulation determines the optimal joint locations between the TFN and stent frame. *In vitro* experimental test qualitatively demonstrates the mechanical flexibility of the flow-diverter with multi-modal bending. *In vivo* micro X-ray and histopathology study demonstrate that the TFN can be conformally deployed in the curved blood vessel of a swine model without any significant complications or abnormalities.

A cerebral aneurysm is a sporadically acquired lesion on a blood vessel that is gradually filled with blood, which causes pressure on a nerve or surrounding brain tissues^1^. Adult population living with a cerebral aneurysm is estimated ~10 million in the United States^2^, indicating a prevalence between 1 and 6%^3^. While most of the aneurysms are not ruptured during the course of a person’s lifetime, the reported annual rupture rate is ~1%^4^. Cerebral aneurysm rupture carries an extremely high rate of morbidity and a mortality rate of up to 50%^2^. Endovascular therapy using platinum coils to fill the aneurysm sac has replaced an open craniotomy for clipping as the most common treatment modality due to its proven superiority in select patients^5^,^6^,^7^,^8^. While endovascular embolization is less invasive and carries a lower treatment risk, the cure rates lag behind those of clipping, primarily due to a significant rate of post-operative recanalization or regrowth due to persistent blood flow into the aneurysm in up to 38% of patients^9^.

Recently, a more efficacious treatment technique has been proposed, which is the placement of a porous mesh stent (i.e. ‘flow-diverter’) adjacent to the cerebral aneurysm. The neurovascular flow-diverter is similar to a typical metallic mesh stent, but it has a much denser array of pores to reduce blood flow. The porous structure maintains the outflow in the side branches and perforators located adjacent to the aneurysm^10^. Clinical outcome with flow-diverters showed that aneurysmal complete occlusion rate is 76% and procedure-related morbidity and mortality are only 5% and 4%, respectively^11^. A flow-diverter is typically collapsed into a neurovascular delivery catheter. After insertion into a blood vessel, this tube is deployed into a larger diameter (~3 mm). The metal film undergoes extreme deformation of 400–500%, which can easily cause the mechanical fracture. Our prior work^12^ in 2011 introduced a microfabrication method to design a hyper-elastic flow-diverter based on a shape memory alloy: thin film nitinol (TFN). While there are several mechanical studies^13^,^14^,^15^,^16^ about stent materials including the TFN, there is no comprehensive study about the mechanical behavior and safety of the TFN, especially for the use as a neurovascular flow-diverter in curved arteries.

In this paper, we fabricate a microstructured TFN membrane and investigate the mechanical safety of the structure by using an *in vitro* experimental model and *in vivo* swine model. Figure 1 shows the overview of a highly flexible TFN device, designed by mechanics modeling and simulation, and demonstration as a flow-diverter in a curved blood vessel (animal test). We focus on the study of the mechanical characteristics of a hyper elastic TFN membrane via computational mechanics modeling, which is validated by quantitative experimental study using a bending and stretching tester. The computational results show that the TFN can be elastically stretched both in radial and longitudinal directions, which are compared to the experiment. The bending mechanics study reveals the extremely flexible characteristics of the TFN. The computational study also captures the underlying physics associated with the optimal attachment of the TFN membrane onto a neurovascular stent backbone, which allows the successful integration with the minimal mechanical loadings on the structure. *In vitro* experimental test results show the TFN flow-diverter qualitatively agrees well with the computational results in multi-modal bending and stretching evaluations in the artificial blood vessel. Both micro X-ray and histopathology results confirm the well-deployed device in the curved blood vessel with a great wall apposition.

## Results

### Finite element modeling

**Figure 2:**
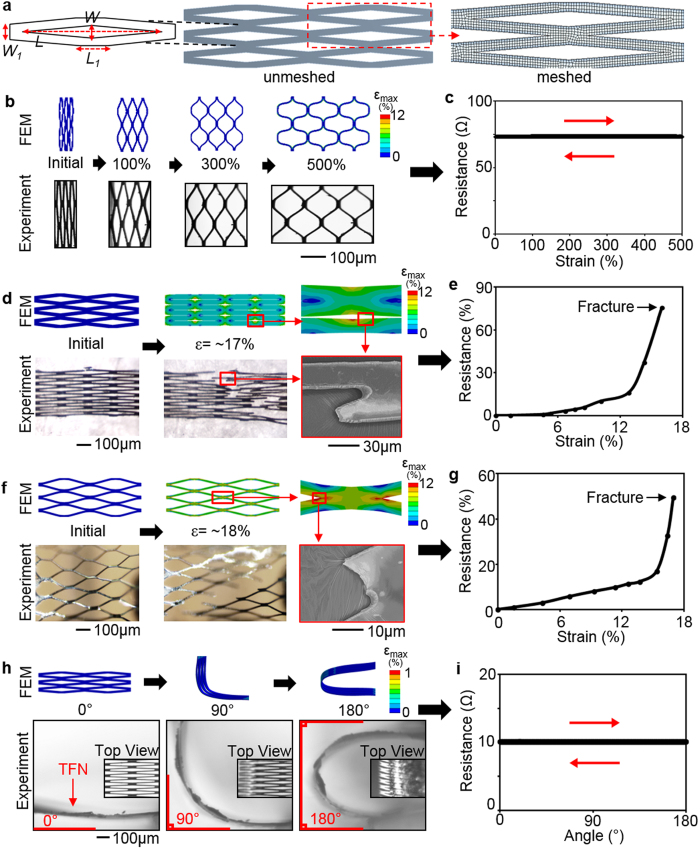
Mechanical behavior of a TFN. (**a**) A geometric model of a TFN with dimensions and meshed structure; lateral length: *L* = 300 μm and *L*_*1*_ = 56 μm and vertical length: *W* = 20 μm and *W*_*1*_ = 24 μm. (**b,d,f,h**) Comparison between the FEM results and optical and scanning electron microscopic images of a TFN for radial stretching (**b**), axial stretching (**d**), biaxial stretching (**f**), and bending (**h**). (**c,e,g,i**) Plot of electrical resistances of a TFN according to the applied strains (**c,e,g**) and the bending curvature (**i**).

3D computational study uses a set of simplified geometric models of a microstructured TFN membrane (Fig. 2a). The finite element modeling estimates the mechanical behavior of the structure upon uniaxial stretching in radial and longitudinal directions, biaxial stretching, and bending. The design focus of the TFN is to resist multi-modal large strains from the mechanical deformation when it is deployed in a blood vessel. We utilized a simulation software (ANSYS Structural Analysis Package, Canonsburg, PA), with user-defined material properties for the TFN membrane, to calculate the stress and strain fields^17^. The governing equation for structures in static equilibrium can be expressed by:


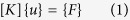


where [*K*] is the stiffness matrix defined by the material properties^18^, {*u*} is the nodal displacement vector, and {*F*} is the associated load vector. In the modeling, we solved this set of simultaneous equations on different nodes with the defined material properties and boundary conditions. To reduce the computational complexity, only a small fraction of the TFN membrane structure (6 repeated patterns) was modeled (Fig. 2a). The diamond pore structure has lateral (*L*) and vertical length (*W*) of 300 μm and 20 μm, respectively. The strut has lateral length (*L*_*1*_) of 56 μm and vertical length (*W*_*1*_) of 24 μm with the membrane thickness (*t*) of 6 μm (Fig. 2a). In order to express the super-elasticity of the TFN, the user-defined shape memory alloy was employed. Table 1 summarizes all parameters used in the modeling^19^. The fracture limit^20^ of the maximum principal strain for the TFN was 12%.

### TFN device fabrication

A hyper-elastic TFN was fabricated by using the “hot-target” DC magnetron sputter deposition and lift-off microfabrication process^12^,^21^. We followed the previously designed method and micropatterning process for the TFN fabrication^12^. After the microfabrication, the surface of the TFN structure was treated by using a hydrogen peroxide to make the surface super-hydrophilic, which provided a great hemocompatibility for *in vitro* and *in vivo* testing^22^,^23^.

### Mechanical behavior of a TFN membrane

A set of mechanical testing of the TFN focused on the applicability of the micro-structure for curved arteries. Figure 2b,c present the computational and experimental results when the TFN is stretched more than 500% by a mechanical stretcher (Supplementary Fig. S1a,b). Both modeling and experimental data present that the TFN has a negligible effect during uniaxial stretching in the radial direction, which was proved by a highly accurate, electrical resistance measurement with the hysteresis testing (relative resistance change: <0.5%). The electrical resistance is related to mechanical properties and integrity of the overall TFN structure. In experiment, different TFN samples have variations of an initial electrical resistance due to different sample volumes and electrical contact points. The calculated maximum principal strain^24^ of the TFN with the tensile strain of 500% is only 11.5% (fracture limit: 12%) and the structure shows no fracture in the experiment (Fig. 2b). The same TFN device revealed a hyper-elastic mechanical behavior upon multiple loading-unloading tests without noticeable change, observed by an optical microscope and electrical measurement (Fig. 2c and Supplementary Fig. S1c,d). Overall, this result proves that the TFN can be stretched in the radial direction more than 500% and safely deployed in a blood vessel.

Another set of computational study in the longitudinal (axial) direction shows the maximum applicable strain of ~17% where it reaches the maximum principal strain value (Fig. 2d). The experimental results, studied by an optical microscope and scanning electron microscope (SEM), show a good agreement with the modeling by revealing the mechanical fracture at the strut junctions. The relative resistance measurement upon the strain change (Fig. 2e) verifies the mechanical fracture at ~17%. Three sets of tests demonstrate the mechanical response of the membrane according to the applied strains are consistent with mild increase upto ~12% and steep increase until completely fractured (Supplementary Fig. S2). Over the transition point, the TFN structure seems to experience a localized necking and fracture. Experimental variations of the alignment and integration of the TFN on the mechanical stretcher caused the difference of the slope and resistance values in multiple tests.

The biaxial mechanical study (Fig. 2f,g and Supplementary Fig. S3) supports the uniaxial mechanical behaviors of the TFN, shown in Fig. 2d. The testing membrane was pre-stretched from ~300% to ~360% in the radial direction and an additional strain in the axial direction was applied to the structure with the increment of ~2%. As long as the pre-loading was within the radial strain limit of the TFN (~500%), the amount of pre-loading did not directly affect to the amount of variations in axial stretching. Overall results show a great agreement with those from the uniaxial study, meaning that the pre-loading in the radial direction is negligible as expected and the mechanical failure is governed by the axial stretching. This experimental study reveals the structural safety of the TFN considering the direct integration with a conventional backbone stent to form a cylindrical flow-diverter.

In addition, a series of the mechanical bending tests was conducted to estimate the membrane integrity when the TFN is deployed in targeted, curved arteries (Fig. 2h,i). The electrical resistance of the structure was measured when the curvature changed from 0 to 180 degrees. Both computational and experimental results show that multi-modal bending does not cause any noticeable mechanical burdens on the structure. The calculated maximum principal strain value in the modeling is less than 1% and the electrical resistance varies less than 0.2% based on the repeated mechanical tests (Supplementary Fig. S4). The results present that the extremely compliant TFN is capable of undergoing excessive bending in the deployment and navigating the highly tortuous nature of neurovascular veins.

### Determination of the optimal junctions for a TFN flow-diverter

**Figure 3:**
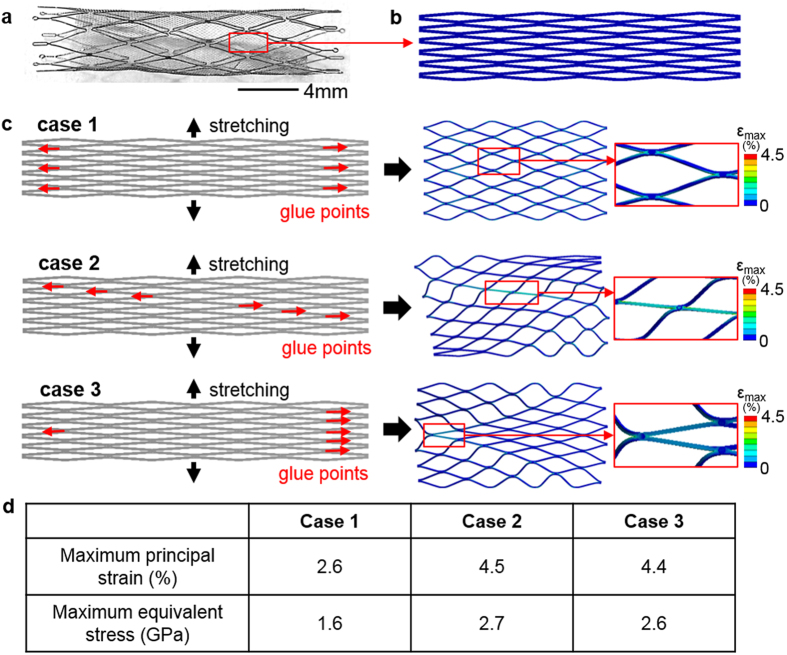
Determination of the optimal junctions according to the attachment patterns. (**a**) Optimal microscopic image of a TFN flow-diverter. (**b**) A TFN geometry for the FEM study. (**c**) Three representative patterns of glue points (six locations) with the vertical stretching for integration with a backbone stent. (**d**) Table summarizing the maximum principal strain and maximum equivalent stress; case 1 shows the lowest values in both criteria.

Figure 3 summarizes the evaluation of attachment patterns between a TFN and stent backbone, based on the values of maximal equivalent stress and maximum principle strain. A flow-diverter includes a TFN membrane and a stent backbone with several attachment locations (Fig. 3a). When deployed in the curved arteries, the hyper-elastic TFN conforms to the geometry of the blood vessels. In this computational study, we modeled a TFN (24 repeated diamond patterns) with several attachment junctions (Fig. 3b). The simulation model includes meshes with 303, 260 nodes and 157, 436 elements and shows a high quality of the meshed elements (Supplementary Fig. S5). The TFN membrane has 200% pre-strains in the radial direction. The faces of attachment locations are constrained in the longitudinal direction to simulate the attachment of the TFN on the stent backbone. We compared three representative patterns of glue joints to determine the optimal pattern when the same loading was applied on the vertical direction to stretch the TFN structures (Fig. 3c). Case 1 considers six glue joints in the vertical direction on both sides, which results in 2.6% of the maximum principal strain (MPS) and 1.6 GPa of the maximum equivalent stress (MES). Diagonal (case 2) and uneven (case 3) glue points show 4.5% (MPS), 2.7 GPa (MES) and 4.4% (MPS), 2.6 GPa (MES), respectively. As summarized in the table (Fig. 3d), case 1 shows the lowest MPS and MES compared to other cases. Thus, we choose the evenly distributed, symmetric glue patterns that provides the highest structural safety upon the given loading condition.

**Figure 4:**
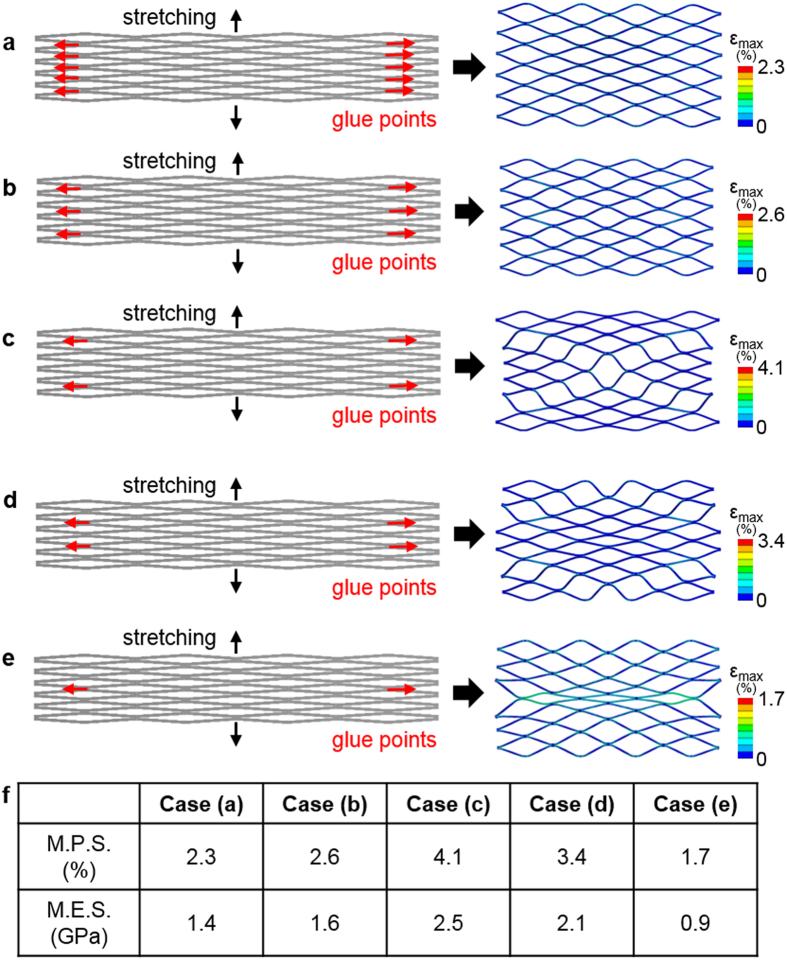
Determination of the optimal junctions according to the number of glue points. (**a–e**) Five different cases with different numbers of glue locations and their FEM results that show the deformation patterns and the maximum principal strains. (**f**) Table summarizing the maximum principal strain (MPS) and maximum equivalent stress (MES); case (**e**) shows the lowest MPS and MES.

In addition to the determined glue pattern, we conducted another set of computational study to find out the effect of the number of glue points to the structure. Figure 4 shows five different cases with varying glue points from 10 to 2. To analyze the mechanical behavior, the same loading was applied to the structures vertically and the MPS and MES were compared. Figure 4a–e show the computational results with the applied loading and Fig. 4f summarizes the values of MPS and MES for five cases. The testing result suggests to minimize the number of glue points for better structural safety upon the mechanical stretching; two symmetrical glue points show only 1.7% of MPS and 0.9 GPa of MES. Following the result, we fabricated a TFN flow-diverter with the minimal use of joining between the microstructured TFN and backbone stent frame, which was used for qualitative *in vitro* mechanical test and *in vivo* feasibility study.

### *In vitro* study of a TFN flow-diverter

**Figure 5:**
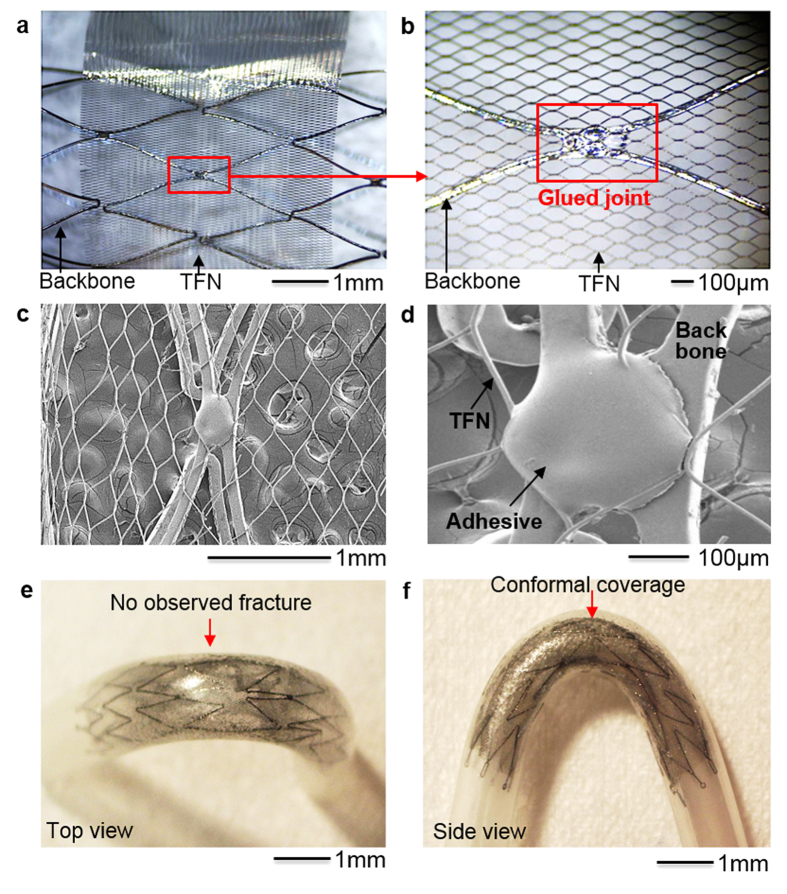
*In vitro* study of a TFN flow-diverter. (**a**) Optical microscopic image of a TFN membrane wrapping a commercial stent backbone. (**b**) Magnified view of the glued junction between the TFN and stent backbone. (**c,d**) Scanning electron microscope images of the assembled flow-diverter having the stent backbones with the microstructured TFN, integrated by using a polymer adhesive. (**e**) Photo (top view) of a TFN flow-diverter deployed in a curved artery model using a delivery catheter system. (**f**) Side view photo of (**e**) that shows the conformally deployed device in the curved model without any significant mechanical fracture.

Figure 5a,b show a prototype TFN membrane that conformally wraps a commercial stent backbone (Neuroform, Stryker Neurovascular, Fremont, CA) using a medical grade, cyanoacrylate instant adhesive (MG30, Adhesive Systems, Frankfort, IL). The TFN device was treated with a hydrogen peroxide^22^, which offered a hemocompatible super-hydrophilic property. A mechanical clamping system with a pair of magnets helps the fabrication process by stretching the TFN to fix on the stent frame (Fig. 5a). A biocompatible adhesive, dropped by a fluid dispenser, makes the optimal junction between the TFN and backbone stent frame as shown in Fig. 5b. Scanning electron microscopy investigations (Fig. 5c,d) show that the polymer adhesive conformally encapsulates the TFN struts on the backbone stent frame.

Afterwards, we conducted a set of qualitative, *in vitro* testing to demonstrate the device deployment using a delivery catheter system with a neurovascular stent (3.0–4.0 French; Fr). Figure 5e,f represent the top and side view of the deployed device in the curved artery model. The testing model was manufactured by using a heat shrink tubing made of polyvinylchloride. The tube was filled with fine powder of sand and highly packed using a vibrator. Then, heat was gradually applied while the tube was underwent the bending with the external forces. Once the tube was curved in an appropriate geometry, the heat was removed and cooled down for 1 minute. After eliminating all sand powder and subsequent cleaning, the tube was used for *in vitro* qualitatively deployment tests. The collapsed devices were delivered in the middle of the curved region and were deployed by pushing the device from the catheter. The evaluation criteria included wall apposition, observance of fracture, and any other issues associated to the collapsing and deployment. This qualitative evaluation reveals that the device could conformally deployed in the vessel with the self-expanding mechanism^25^,^26^. The device makes 400% radial stretching in the 3 mm-diameter artery model. None of mechanical fracture of the device or ruptured films was observed through the microscope investigations, which represents a good agreement with the computational modeling results in the stretching behavior of the TFN. This result shows the potential of the TFN flow-diverter for the clinical use in a curved, tortuous neurovascular system that involves severe longitudinal elongation of the TFN structure due to the bending when deployed.

### *In vivo* study of the device feasibility with a swine model

**Figure 6:**
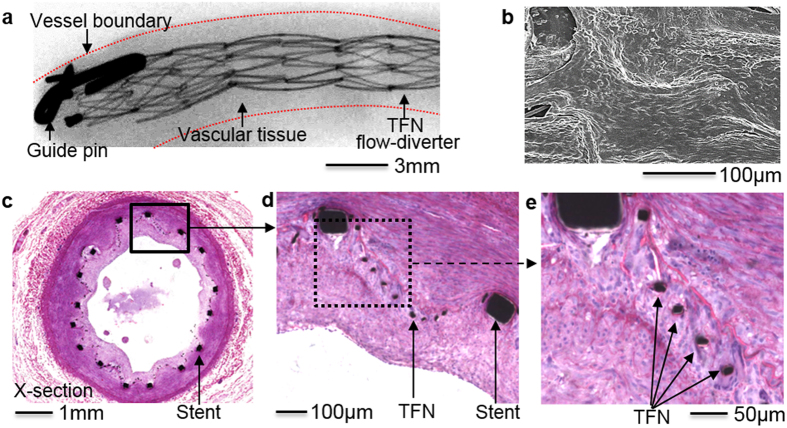
*In vivo* study of the device feasibility. (**a**) Micro X-ray image (30 days follow-up) of a TFN flow-diverter deployed in the distal region of the right common carotid artery in a swine model. (**b**) Scanning electron microscopy image showing the device with the newly grown healthy endothelial cell layers. (**c**–**e**) Histopathology images of the flow-diverter implanted over a month. The results show nicely grown tissues on the highly porous, hyper-elastic TFN membranes without significant thrombosis formation or abnormalities.

Proof-of-concept, *in vivo* study using a swine model made an assessment of both the functionality and biocompatibility of the prototype TFN device. Figure 6a represents a micro X-ray image of the nicely deployed, TFN flow-diverter in the distal region of the right common carotid artery in a swine after 30 days of the insertion. In the monochromatic image, the microstructured TFN is not visible, but only the macro-scale, backbone stent frames due to the limited resolution of the typical medical grade fluoroscopy. Overall, the flow-diverter shows a well-deployed device in the slightly curved blood vessel with some irregular tissue in-growth due to the non-uniform growth of the vascular cells (e.g., smooth muscle and endothelial cells) through the microstructured TFN. Figure 6b shows a scanning electron microscopy image after one month of the device deployment that has healthy endothelial cell layers to cover the luminal surface of the blood vessel, which form a confluent endothelial layer with cobble stone patterns. The newly grown tissues entirely covered the microstructured TFN with no significant platelet or other blood products attachment, which is an ideal outcome of the device implantation in the vascular system. A few of the TFN struts are visible on the surface because of the supercritical drying process to fix the tissue. A histopathology image (Fig. 6c) shows that some degree of neointimal tissue growth (i.e., new grown tissue through the device) over a month after TFN flow-diverter implantation. This tissue growth could be caused by the hyper-elastic TFN in this test had a lateral dimension of 500 μm, which had almost 300 μm × 300 μm fenestrations after the device deployment in the artery. Smooth muscle cells could easily travel through the micro fenestrations of the TFN flow-diverter because of the size (typically a few tens microns) and spindle-like shapes^27^. The zoomed-in view of the tissue growth (Fig. 6d) captures the highly porous TFN membranes. The grown tissue from the wall to the lumen relocates the TFN struts because the TFN is hyper-elastic and easily elongated. Additionally, Fig. 6e shows biological response of the vascular wall with the TFN flow-diverter though the pathological study. This *in vivo* results demonstrate that the TFN flow-diverter has no significant thrombosis formation or abnormalities in the swine model, which shows the potential of the device for applications in the treatment of various types of vascular aneurysms including neurovascular aneurysm. The *in vivo* test performed in this work was only intended to present a proof-of-concept for the use of a microstructured TFN as a neurovascular flow-diverter. Currently, we plan to have more thorough *in vivo* tests using various TFN coverings for the study of the device efficacy and safety.

## Discussion

The microstructured TFN, optimally assembled with a conventional stent backbone, enables a successful deployment in curved blood vessels without mechanical fracture and plastic deformation. The computational mechanics modeling and simulation reveals the excellent mechanical bendability, flexibility, and stretchability of the TFN and a series of experimental tests including uniaxial stretching in radial and axial directions, biaxial stretching and bending show well-agreed results with the computational study. Additional computational modeling make the ideal and optimal assembly between the TFN and backbone stent possible as a flow-diverter. The feasibility of the fabricated device is supported by both *in vitro* and *in vivo* studies; qualitative microscopic observation shows no significant plastic deformation and mechanical fracture in a curved artery model and hemocompatibility and histopathology based on a swine model supports the qualitative study by showing no thrombosis formation and abnormalities. Overall, the microstructured TFN device shows the potential as a flow-diverter in various aneurysms, specifically challenging neurovascular applications. Areas for further development include three-dimensional (3D) computation that allows modeling and simulation of various types of TFN designs and complicated structures integrating two or more backbone structures together as a neurovascular flow-diverter. A 3D structured flow-diverter model could enable more sophisticated analysis of the mechanical behavior of the TFN device when deployed into various types and shapes of curved arteries and blood vessels. In addition, an automated mechanical tester having a set of accurate linear motors would offer more accurate measurement of the mechanical (bending, radial, and stretching) behaviors of the entire devices and structures. Additional functions could be included in the TFN device such as flow and pressure monitoring microsensors, which would enable an active, quantitative assessment of the time-dynamic treatment process of aneurysms.

## Conclusions

The microfabricated TFN membrane as a flow-diverter shows an extreme mechanical bendability (up to 180°) and stretchability (>500%) in the radial direction. The comparison between the computational and experimental studies based on uniaxial, biaxial, and bending mechanics show the very good agreement, which proves the mechanical stability of the TFN microstructure upon multi-modal mechanical deformations. A prototype device, fabricated based on the simulation result of the optimal assembly of the TFN with a backbone stent, demonstrates the feasibility as a flow-diverter through *in vitro* experiment and *in vivo* swine model test; qualitative *in vitro* model shows the device has an excellent conformality when deployed in a modeled vessel without any mechanical fracture, while *in vivo* study with a swine model proves the successful integration of the TFN device into a curved artery (over a month) without any abnormalities and thrombosis formation. Areas for further development include more accurate 3D computational modeling and incorporation of active sensing components for real-time monitoring of intra-aneurysmal hemodynamics in the blood vessel.

## Methods

### Computational modeling

For radial loading, the TFN structure was modeled with purely Hex20 elements and two symmetric displacement loadings in radial direction were applied on two edges of the structure (Supplementary Fig. S6). The structure surface was constrained from out-of-plane motion to simplify the problem into 2 dimensional motion. The magnitude of the displacement loadings was gradually increased and the maximal equivalent stress as well as maximum principle strain fields were calculated to determine the potential fracture locations in the structure. Here, we defined the strain % as the displacement loading over the original distance and assumed that the fracture occurred on the location where the maximum principle strain exceeded the strain limit of the nitinol material. The TFN structure was modeled with a combination of Hex20 and Wed15 elements in longitudinal (axial) loading analysis (Supplementary Fig. S7). Similarly, two symmetric displacement loadings in axial direction were applied on two edges to stretch the TFN membrane out in horizontal direction. We gradually increased the magnitude of the displacement loading and the maximal equivalent stress as well as maximum principle strain field was computed to identify the fracture locations. Supplementary Fig. S8 describes the details for modeling of the biaxial stretching. In first step, a 100% strain displacement was applied on two faces on the top and other two on the bottom. This is the step where the TFN structure was stretched in the radial direction before being wrapped around a stent backbone. We only applied 100% strain displacement to minimize the buckling issues, which will increase the computation complexity^28^. Supplementary Fig. S9 presents the modeling results of the mechanical bending. To analyze the mechanical behaviors of the TFN structure subject to deployment in curved vessels, a three-point bending analysis was performed with a combination of Hex20 and Wed15 elements.

### Experiment of uniaxial stretching

A small rectangular sample of TFN was cut and affixed to a custom-made mechanical stretcher^29^,^30^. Two separate uniaxial loading tests were performed; one in the radial direction, and one in the longitudinal direction. For the radial loading, the sample was aligned vertically along the length of the stretching machine such that strain was only applied widthwise along the diamond fenestrations (Supplementary Fig. S1). For the longitudinal loading, the sample was aligned horizontally along the length of the stretching machine such that the strain was only applied lengthwise along the diamond fenestrations (Supplementary Fig. S2). The stretching machine had one fixed support block (slide) with a copper strip running down its length, the sample was glued to the copper and colloidal silver paste was applied to secure electrical conductance. The other side of the sample was affixed to an adjustable support block (slide) in the same fashion. The copper strips were connected to wires so that the electrical resistance of the sample could be measured via a digital multimeter (Model 2100, Keithley, Cleveland, OH). An initial resistance measurement was taken while the sample was at rest (0% strain) for a baseline. Resistance of the sample was recorded to document the structural integrity of the material whereas an increase of resistance would indicate structural degradation of the sample. The adjustable slide was then moved in approximately 2% increments to increase the distance between the two slides, thereby increasing the strain on the sample. The percentage of the strain was calculated by the percentage increase of distance over the original distance. The increase in resistance compared to the increase in strain was recorded and plotted for each increment until the mechanical failure of the sample. After the first fracture point on the sample occurred, the structure was investigated by scanning electron microscopy.

### Experiment of biaxial stretching

The samples were placed under preloaded strains ranging from ~300% to ~360% along the width (in the radial direction) and then connected to two separate electrical wires (Supplementary Fig. S3). The samples were wrapped circumferentially around the wires to mimic the shape of the stent backbone. Similarly to the uniaxial sample, it was glued at every point around the wire and electrical conductivity was ensured using colloidal silver paste. One wire was attached to the fixed slide, and the other attached to the adjustable slide. The testing then proceeded the same way as for the uniaxial sample.

### Bending test

Using the same sample setup as the uniaxial test, the slides were instead attached to a bending machine where one slide is fixed and the other is able to rotate the sample from 0° to 180° (Supplementary Fig. S4). The center of rotation was located slightly above the sample to allow for soft bending (“U” shape at 180°) which corresponds to neurovascular patterns. An initial resistance measurement at rest (0°) was taken as a baseline for the test. The rotatable slide was then steadily moved from the 0° position to the 180° position and then back to the 0° position, while the resistance was continuously monitored and recorded. Optical images of the sample were taken at 0°, 90°, and 180° by using a portable USB microscope (Dino-Lite, Torrance, CA)^30^.

### *In vivo* study using a swine model

A proof-of-concept *in vivo* test with a swine model was carried out in accordance with the IACUC approved protocol (#2007-129-11, University of California, Los Angeles Chancellor’s Animal Research Committee). A female Yorkshire pig (30–40 kg) was used for the study. Under fluoroscopic guidance the device was deployed in the distal region of the slightly curved right common carotid artery. The repeat angiograms were performed at 30 days, then, the deployed device was harvested and stored in a solution of 10% formaldehyde for tissue fixation for further micro X-ray and histopathology analyses.

## Additional Information

**How to cite this article**: Chen, Y. *et al*. Microstructured Thin Film Nitinol for a Neurovascular Flow-Diverter. *Sci. Rep.* **6**, 23698; doi: 10.1038/srep23698 (2016).

## Supplementary Material

Supplementary Information

## Figures and Tables

**Figure 1:**
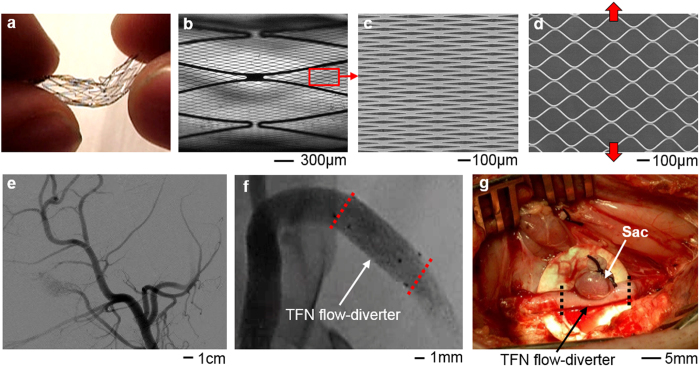
Microstructured thin film nitinol (TFN) for a neurovascular flow-diverter. (**a**) Image of a highly flexible and stretchable TFN. (**b**) Optical microscopic image of the TFN in (**a**). (**c,d**) Scanning electron microscopic images of the magnified view of (**b**); an array of TFN struts (**c**) and the stretched struts in the vertical direction (**d**). (**e,f**) X-ray images of curved blood vessels in a swine model. (**g**) Image of a swine model with a sac and deployed TFN membrane.

**Table 1:** Materials properties of the TFN membrane.

**Parameters**	**Definition**	**Value**
Young’s modulus	Elastic modulus of the material	60 GPa
Poisson’s ratio	Ratio of transverse contraction to longitudinal extension	0.3
Sigma SAS	Starting stress value for the forward phase transformation	52000 psi
Sigma FAS	Final stress value for the forward phase transformation	60000 psi
Sigma SSA	Starting stress value for the reverse phase transformation	30000 psi
Sigma FSA	Final stress value for the reverse phase transformation	20000 psi
Epsilon	Maximum residual strain	0.07
Alpha	material response ratio between tension and compression	0
