# Supplementary material for: Microstructured Thin Film Nitinol for a Neurovascular Flow-Diverter
Source: Sci Rep. 2016 Mar 24;6:23698. doi: 10.1038/srep23698 (PMC4806313; doi:10.1038/srep23698)
Supplement: Supplementary Information [file srep23698-s1.pdf]

Supplementary Information

Microstructured Thin Film Nitinol for a Neurovascular Flow-Diverter

Yanfei Chen[1,+], Connor Howe[2,+], Yongkuk Lee[2], Seongsik Cheon[3], Woon-Hong Yeo[2,4,*] & Youngjae Chun[1,5,*]

[1]Department of Industrial Engineering, University of Pittsburgh, Pittsburgh, PA 15261, USA.

[2]Department of Mechanical and Nuclear Engineering, Virginia Commonwealth University, VA 23284, USA.

[3]Division of Mechanical and Automotive Engineering, Kongju National University, Cheonan, Chungnam, 314-701, Republic of Korea.

[4]Center for Rehabilitation Science and Engineering, Virginia Commonwealth University, VA 23298, USA.

[5]Department of Bioengineering, University of Pittsburgh, PA 15261, USA.

[+]These authors contributed equally to this work.

*Correspondence and requests for materials should be addressed to W.-H.Y. or Y.C. (email: whyeo@vcu.edu or yjchun@pitt.edu)

Supplementary Figure 1

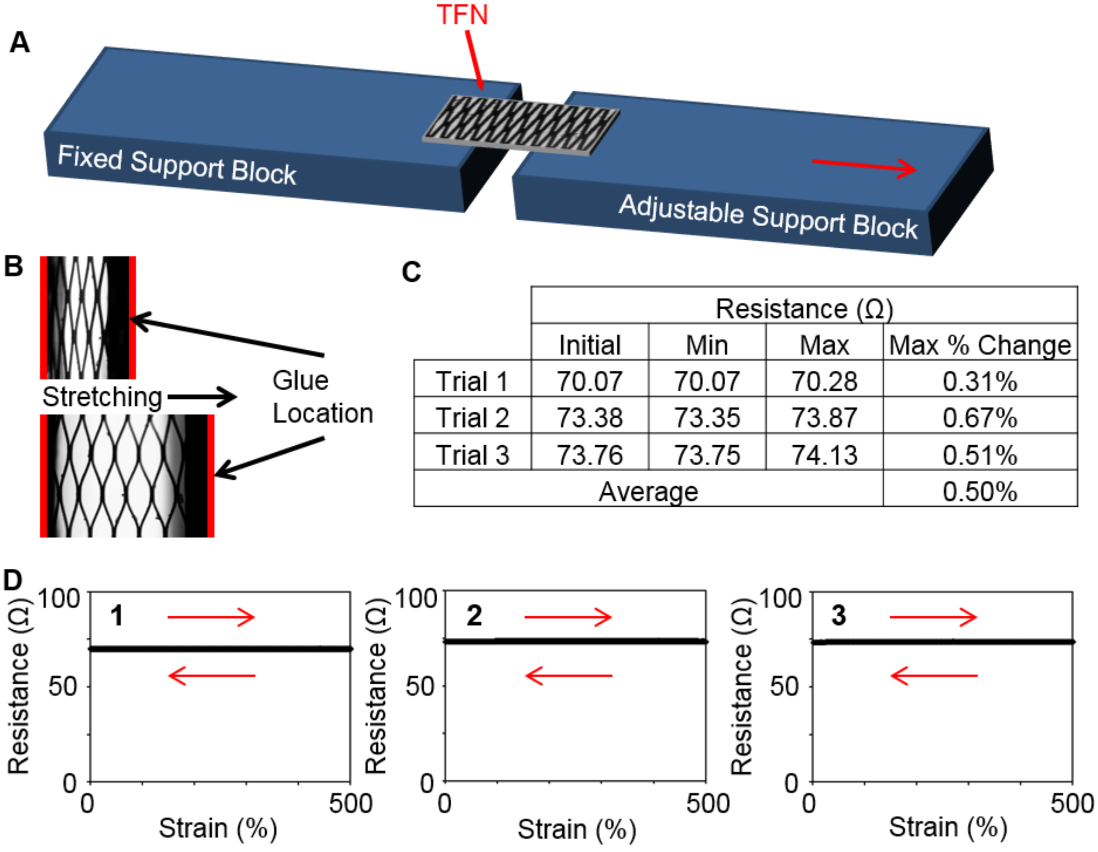

	Resistance (Ω)			
	Initial	Min	Max	Max % Change
Trial 1	70.07	70.07	70.28	0.31%
Trial 2	73.38	73.35	73.87	0.67%
Trial 3	73.76	73.75	74.13	0.51%
Average				0.50%

Figure S1. (a) Experimental setup for mechanical stretching of a TFN in the radial direction. (b) Optical images of a TFN at the initial stage (top) and with the applied strains (bottom). The testing sample was mounted on the mechanical tester at the glue locations (red lines). (c) Summary of the radial stretching of three TFN membranes; values show the electrical resistance change according to the applied strains of 500%. (d) Graphs of three trials of the mechanical testing. Arrows indicate the change of electrical resistance when a TFN was stretched and relaxed.

Supplementary Figure 2

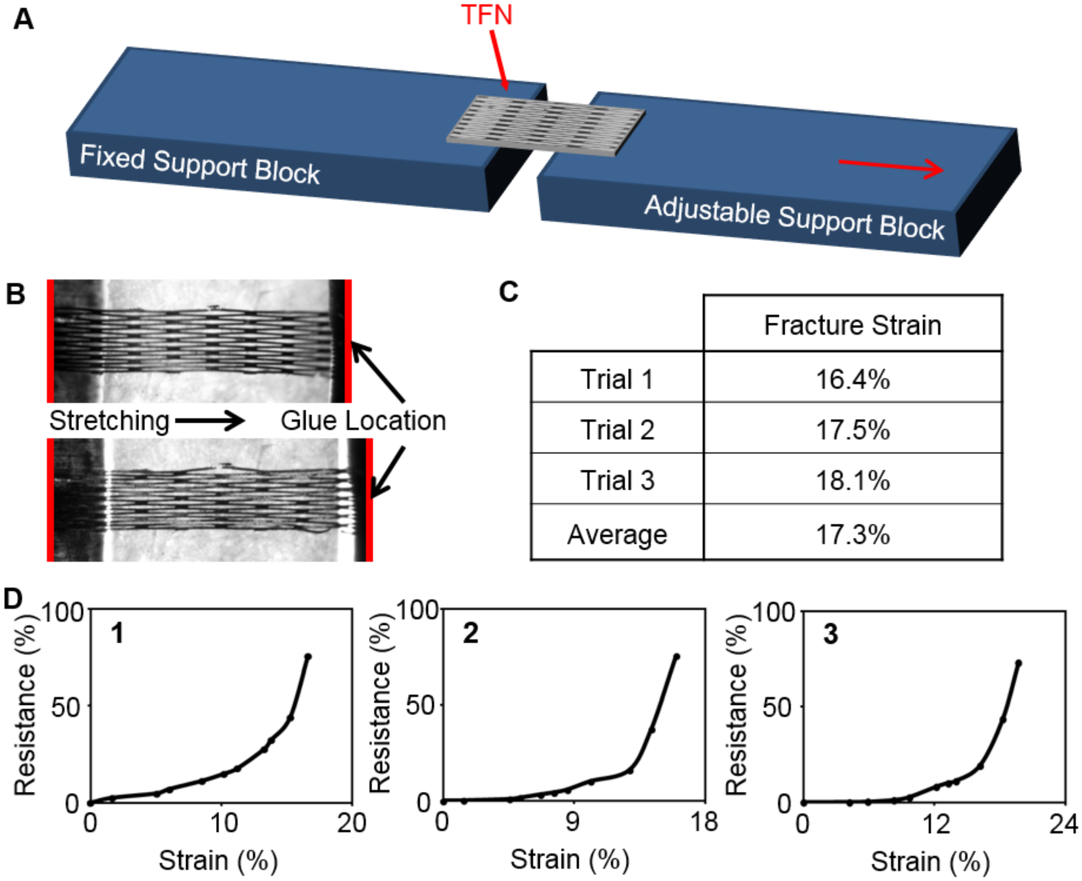

	Fracture Strain
Trial 1	16.4%
Trial 2	17.5%
Trial 3	18.1%
Average	17.3%

Figure S2. (a) Experimental setup for mechanical stretching of a TFN in the longitudinal (axial) direction. (b) Optical images of a TFN at the initial stage (top) and with the applied strains (bottom). The testing sample was mounted on the mechanical tester at the glue locations (red lines). (c) Summary of the longitudinal stretching of three TFN membranes; values show the relative increase of electrical resistance according to the applied strains. (d) Graphs of three trials of the mechanical testing.

Supplementary Figure 3

A

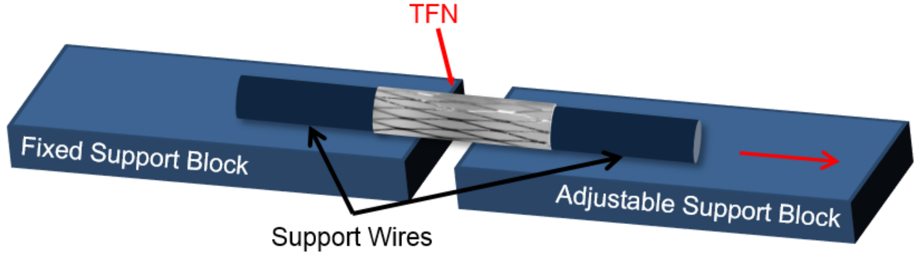

B

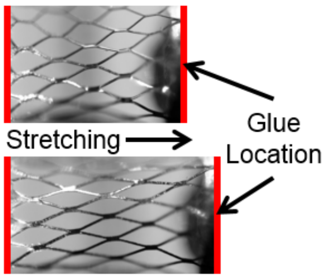

C

	Preload Strain	Fracture Strain
Trial 1	~360%	21.5%
Trial 2	~300%	18.7%
Trial 3	~300%	17.2%
Trial 4	~300%	20.3%
Average		19.4%

D

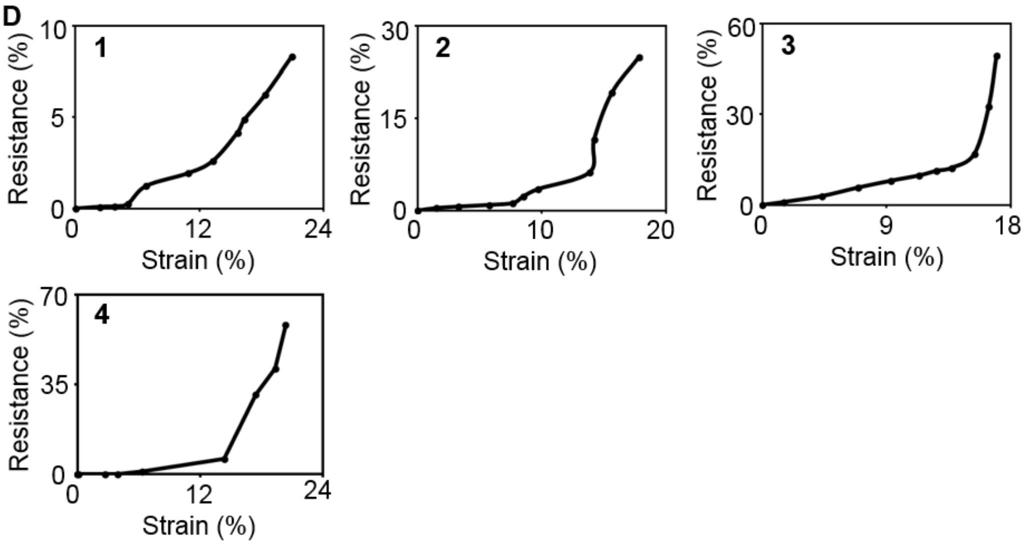

Figure S3. (a) Experimental setup for a biaxial mechanical stretching of a TFN on a cylindrical block. (b) Optical images of a TFN at the initial stage (top) and with the applied strains (bottom). The testing sample was wrapped on a cylindrical block at the glue locations (red lines). (c) Summary of the biaxial stretching of four TFN membranes; preload strain shows the applied strains in the radial direction and fracture strains occur in the longitudinal direction. (d) Graphs of four trials of the mechanical testing.

Supplementary Figure 4

A

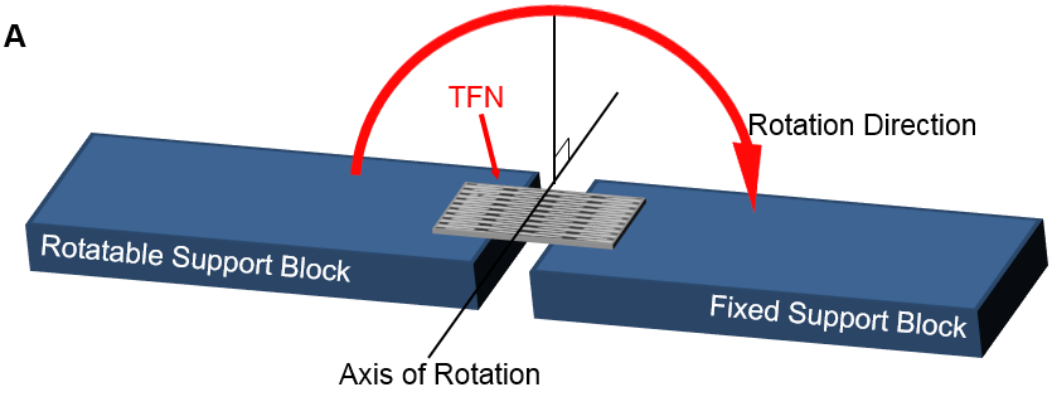

B

Bending Test	
Initial Resistance (Ω)	10.142
Min Resistance (Ω)	10.124
Max Resistance (Ω)	10.163
Change in Resistance (%)	±0.2%

Figure S4. (a) Experimental setup for a mechanical bending test with the rotation from 0 to 180 degrees. (b) Summary of the bending test. The change of electrical resistance is negligible with less than 0.2%.

Supplementary Figure 5

A

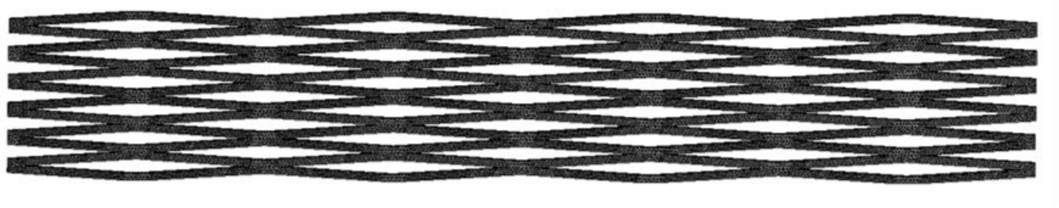

B

Mesh Metric	Element Quality
Min	0.1940
Max	0.9999
Average	0.8630

C

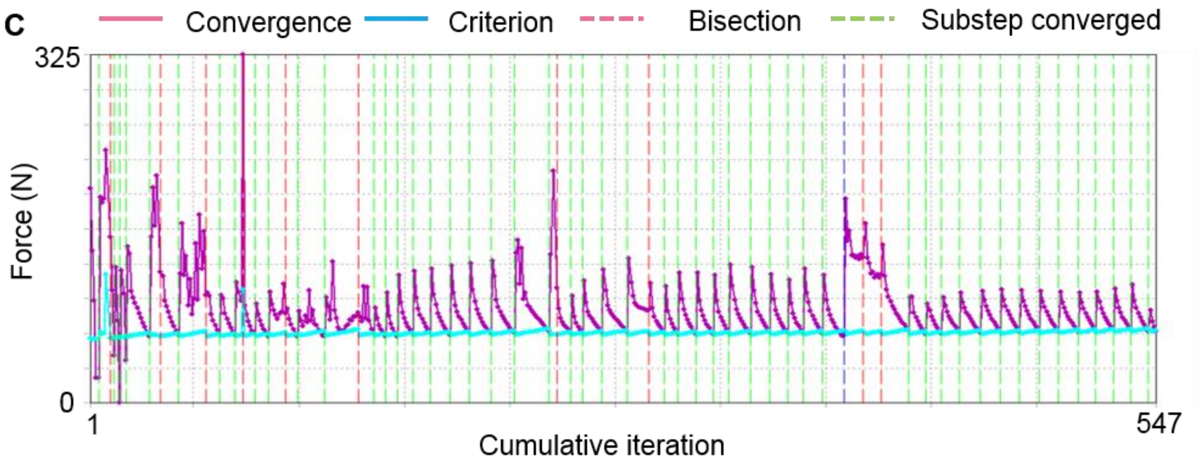

Figure S5. (a) The meshed model for a TFN membrane in ANSYS Static Structural 15.0. (b) The quality of the meshed elements; the value close to 1 represents high quality. (c) Graph of force convergence based on the Newton-Raphson method.

Supplementary Figure 6

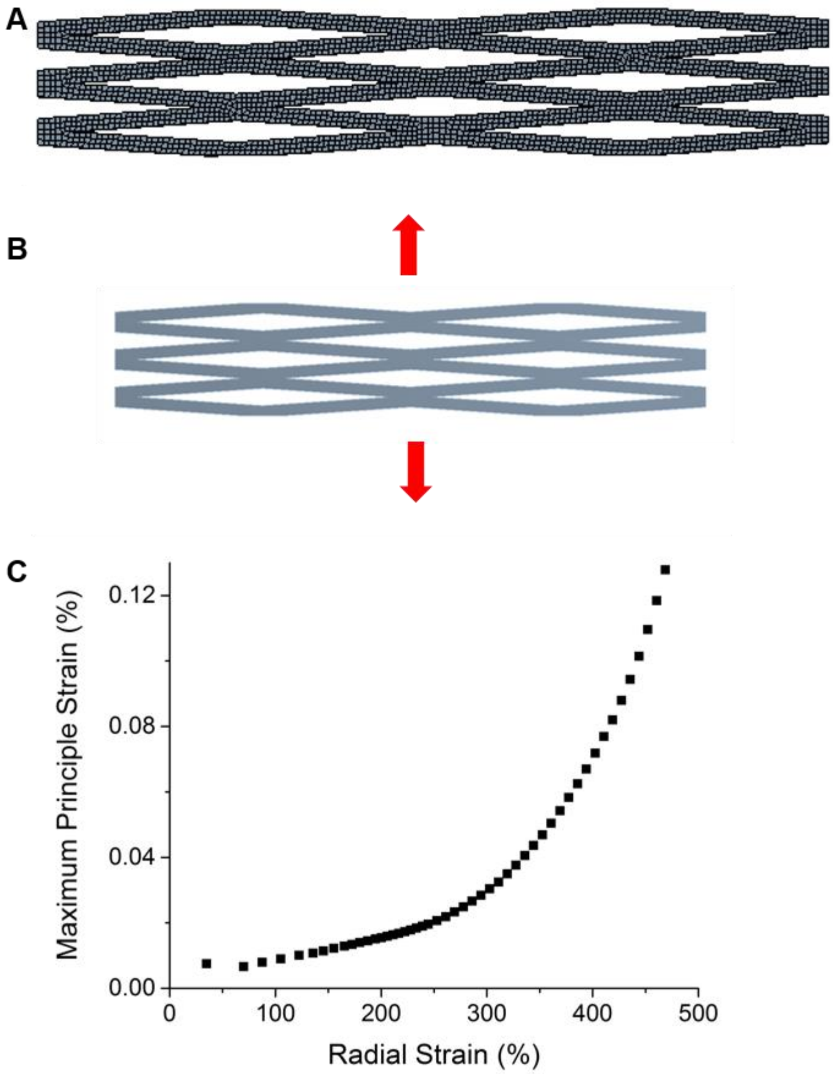

Figure S6. (a) The meshed model for a TFN membrane in ANSYS Static Structural 15.0. (b) Boundary conditions for the modeling; red arrows represent the loading directions (radial direction). (c) The relationship between the calculated maximum principle strains (%) according to the applied strains (%).

Supplementary Figure 7

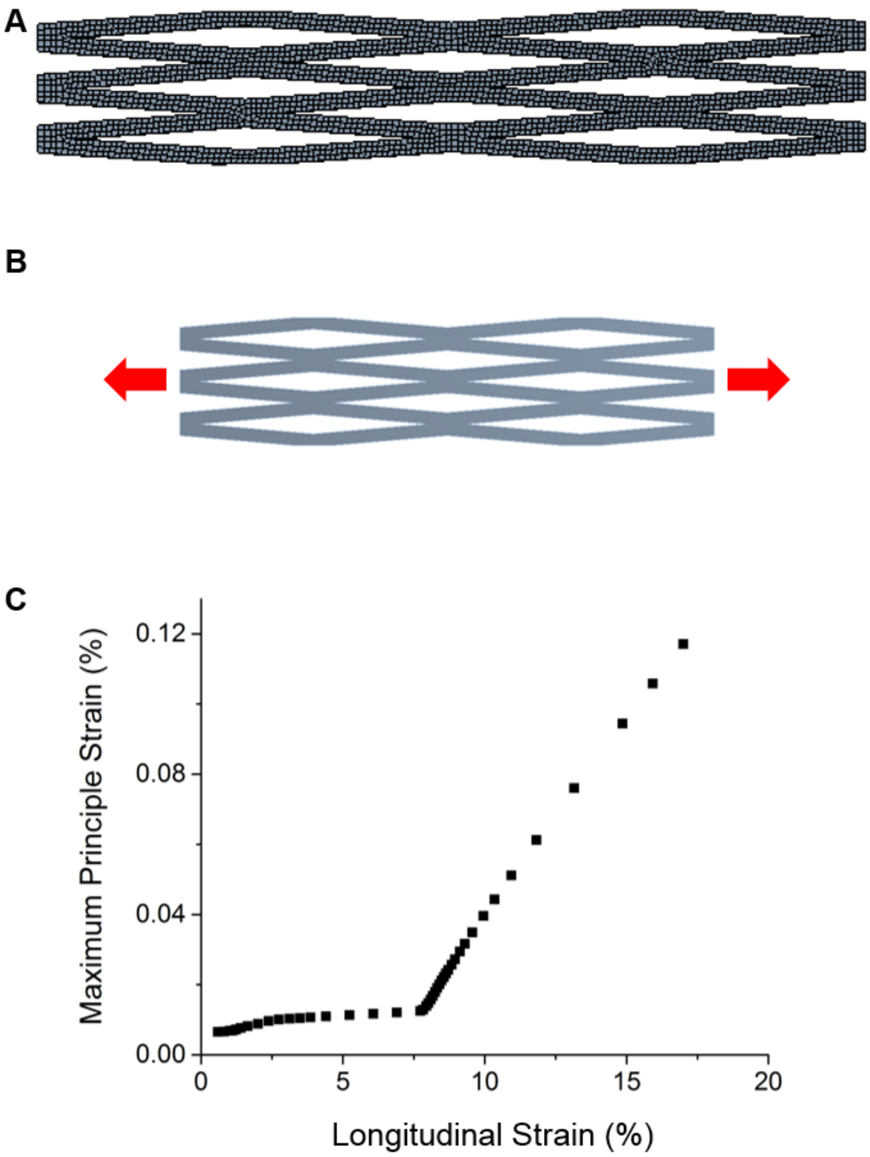

Figure S7. (a) The meshed model for a TFN membrane in ANSYS Static Structural 15.0. (b) Boundary conditions for the modeling; red arrows represent the loading directions (longitudinal direction). (c) The relationship between the calculated maximum principle strains (%) according to the applied longitudinal strains (%).

Supplementary Figure 8

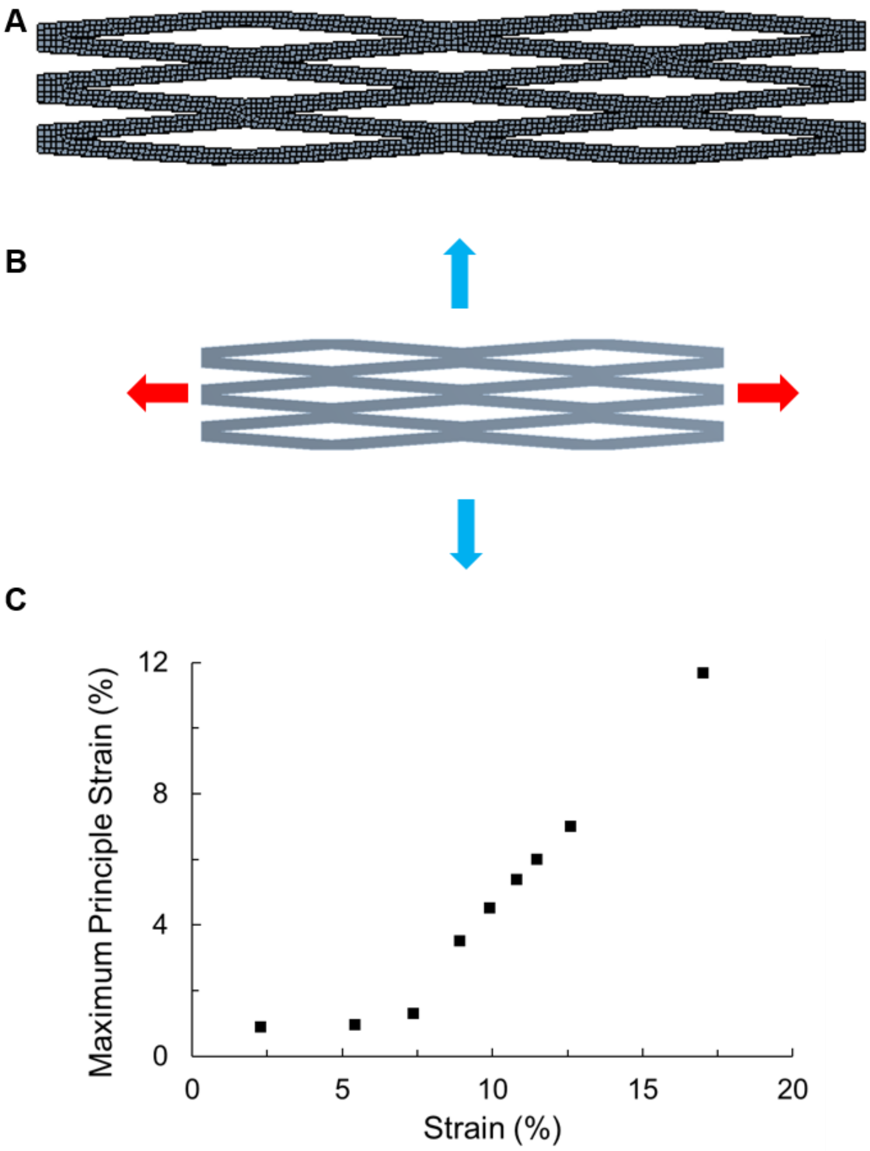

Figure S8. (a) The meshed model for a TFN membrane in ANSYS Static Structural 15.0. (b) Boundary conditions for the modeling; red arrows represent the longitudinal loading, while blue arrows show the radial loading. (c) The relationship between the calculated maximum principle strains (%) according to the applied strains (%).

Supplementary Figure 9

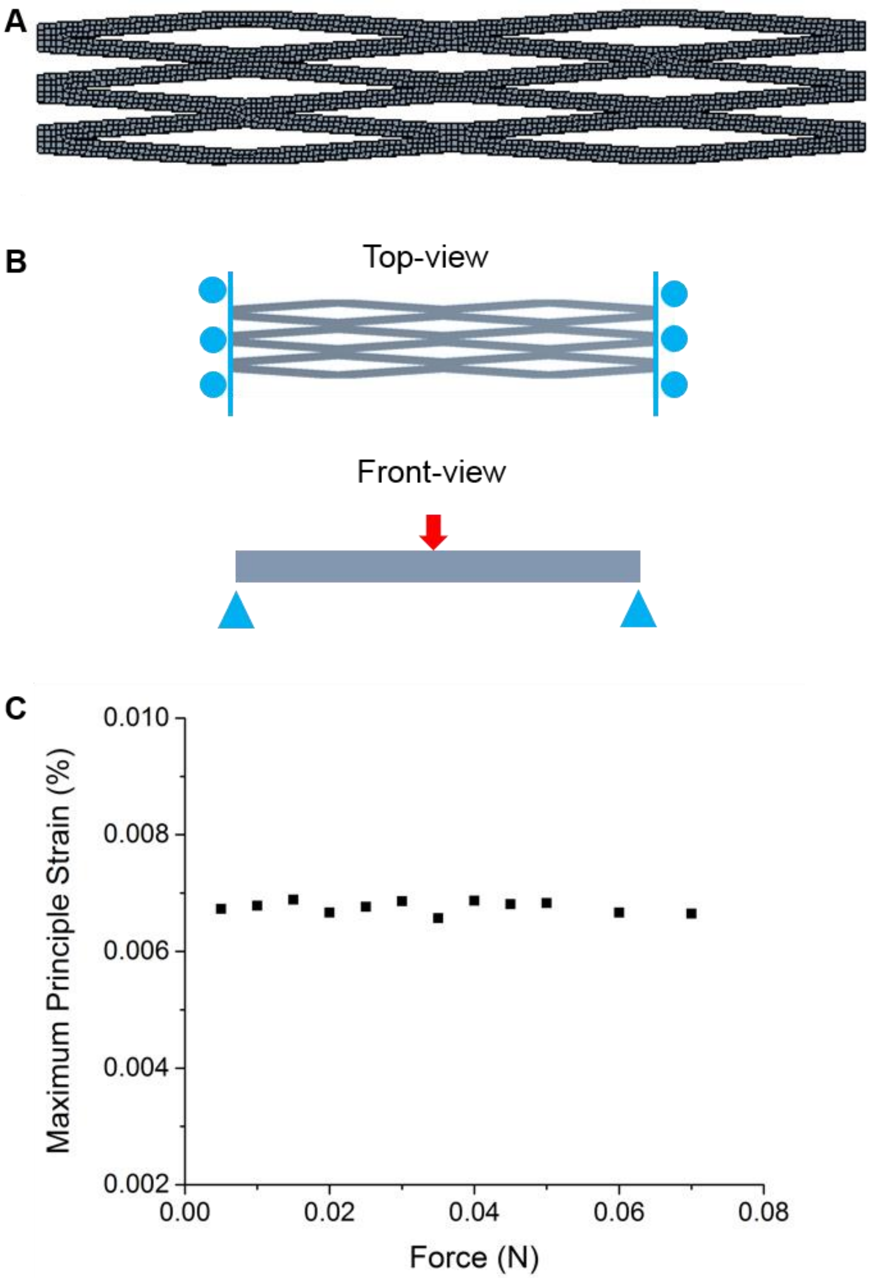

Figure S9. (a) The meshed model for a TFN membrane in ANSYS Static Structural 15.0. (b) Boundary conditions for the modeling; blue rollers in the top view represent the unconstrained motion along the direction, while the red arrow in the front view shows the applied force direction and the blue triangles shows the constraints. (c) The relationship between the calculated maximum principle strains (%) according to the applied forces (N).
